# Diagnostic accuracy and inter-lot precision of the FujiLAM II point-of-care assay to detect tuberculosis in stored urine specimens from people living with HIV

**DOI:** 10.1128/spectrum.00620-26

**Published:** 2026-06-30

**Authors:** Nestor Biatu, Cyrille Mbuli, Comfort Vuchas, Rita Nsamenang, Nadege Bonkar Chifu, Asonganyi Etiendem, Diana Kolieghu Tcheumeni, Angela Neh, Ngha Ndze Mbuh, Nadia Kontogianni, Rachel Owen, Ndi Norah Nyah, Luan Nguyen Quang Vo, Andrew Codlin, Jacob Creswell, Melissa Sander, Ana Isabel Cubas Atienzar

**Affiliations:** 1 Center for Health Promotion and Research, Bamenda, Cameroon; 2 Liverpool School of Tropical Medicine 9655, Liverpool, England, United Kingdom; 3 Cameroon Baptist Convention Health Service 298994, Bamenda, Northwest Region, Cameroon; 4 Friends for International TB Relief, Hanoi, Vietnam; 5 Department of Global Public Health, Karolinska Institute 123913, Stockholm, Sweden; 6 Stop TB Partnership 706700 , Geneva, Switzerland; End TB Dx Consulting LLC (San Diego, US), San Diego, California, USA

**Keywords:** point of care, diagnostics, *Mycobacterium tuberculosis*

## Abstract

The Fujifilm SILVAMP TB LAM II (FujiLAM II) assay is a lateral flow assay that detects TB from urine specimens. This updated version of the assay has been redesigned to address inter-lot variability issues of the original FujiLAM assay. We evaluated the diagnostic accuracy and inter-lot variability of the FujiLAM II assay on biobanked urine specimens collected from inpatients and outpatients living with HIV in two prospective studies conducted at hospitals in Cameroon. Diagnostic performance was evaluated against microbiological reference standards and compared to the Determine TB LAM Ag (AlereLAM) test and smear microscopy. From January to March 2025, urine specimens from 798 people with HIV were tested and analyzed, including 128 (16%) with microbiologically confirmed TB. Against a microbiological reference standard including two sputum liquid cultures and sputum, urine, and blood Xpert Ultra, the sensitivity of the FujiLAM II was 49% (58/119, 95% CI 40%–58%) as compared to 35% (42/119, 95% CI 27%–44%) on the AlereLAM, and the specificity of FujiLAM II was 96% (621/650, 95% CI 94%–97%) as compared to 92% (601/650, 95% CI 90%–94%) on the AlereLAM. Among a subset of 161 participants with any TB result positive, positivity across four manufacturing lots ranged from 37% (60/161, 95% CI 30%–45%) to 42% (67/161, 95% CI 34%–49%), with no significant difference across lots (Cochran’s *Q* = 4.2, *P* = 0.2). These results suggest the FujiLAM II assay has the potential to improve TB diagnosis in some populations, as compared to the only currently available LAM test.

**IMPORTANCE:**

Many people with TB are never diagnosed with and treated for the disease. While sputum-based tests for TB based on molecular diagnostics are highly sensitive, there are many barriers to access due to the high cost and infrastructure requirements of these tests. Accurate, point-of-care tests that can be conducted in peripheral settings without equipment and on non-sputum specimens have the potential to greatly increase access to TB testing and close this gap in care. The Fujifilm SILVAMP TB LAM II test is a new urine-based lateral flow test for TB. Our results show that this test is more sensitive to detect TB in people living with HIV than the only currently recommended urine point-of-care TB test and that the test has consistent performance across lots.

## INTRODUCTION

Many people with tuberculosis (TB) are never diagnosed and treated for the disease, contributing to 1.23 million deaths due to TB in 2024 (1). Current recommendations for TB diagnostics include rapid nucleic acid amplification tests (NAATs) (2) that are highly accurate but are not accessible in many settings due to their cost and infrastructure requirements. Tests that can be performed at the point of care, rapidly and without equipment, have the potential to assist in closing this large gap in the TB care cascade (3).

Mycobacterial lipoarabinomannan (LAM) is part of the *Mycobacterium tuberculosis* cell wall and can be found in the urine of people with TB; LAM has been extensively studied as a biomarker for TB disease (4, 5). In 2015, the World Health Organization (WHO) recommended the Determine TB LAM Ag assay (AlereLAM; Abbott, USA), a lateral flow assay based on the detection of LAM, for the detection of TB in people living with HIV using urine specimens (6). This assay has moderate sensitivity in people with HIV and is recommended to be conducted in parallel with sputum NAAT tests in this population. Another test based on LAM, the Fujifilm SILVAMP TB LAM (FujiLAM; Fujifilm, Japan) lateral flow assay, had potential for use in wider populations, including people without HIV, based on extensive analytical and clinical evaluations (7) but subsequently was shown to have lot-to-lot variability (8, 9).

Recently, a redesigned version of the FujiLAM, the Fujifilm SILVAMP TB LAM II (FujiLAM II), has been developed. The assay was redesigned to address the inter-lot variability and reduce material inputs. In addition, manufacturing was relocated to a low labor cost country for greater cost-effectiveness. Evaluations of the performance of this redesigned assay are urgently needed. The WHO has provided guidance for manufacturers of rapid tests to detect LAM in the form of technical specifications (TSS-23), including for evaluations of diagnostic performance (10).

Here, we evaluated the diagnostic performance of the new FujiLAM II assay on biobanked urine specimens collected from people living with HIV in Cameroon, which is a high HIV-TB burden country (1). We also assessed the lot precision for the assay on a subset of participants who tested positive for TB.

## MATERIALS AND METHODS

### Study design

This was a secondary analysis in which we evaluated the diagnostic accuracy of the FujiLAM II assay using biobanked urine specimens. Specimens were collected from adults living with HIV who had been enrolled in two prospective studies in Cameroon. Participants in the first cohort were enrolled at the admission wards of two hospitals, while participants in the second cohort were enrolled at the outpatient unit and HIV treatment and care centers of four hospitals (cohort details are provided in Table S1, unpublished). These studies were selected because of the availability of stored urine specimens from both inpatients and outpatients, all of whom underwent comprehensive TB evaluations (including AlereLAM testing). Additionally, we compared the diagnostic sensitivity and specificity of FujiLAM II with that of AlereLAM and evaluated inter-lot precision on four different lots of the FujiLAM II assay. The study was conducted to be in line with specifications described in the WHO TSS-23 (section 4.02.03) to contribute to clinical performance studies of rapid assays to detect LAM in urine (10).

All testing was performed at the Tuberculosis Reference Laboratory Bamenda, which is accredited in accordance with the recognized International Standard ISO 15189:2022 (SANAS Accredited Medical Laboratory, no. M0593) over a 3-month period (January–March 2025). This study is reported following the STARD guidelines (11).

### Laboratory procedures

#### Specimen handling, blinding, and randomization

For FujiLAM II testing, frozen urine samples of 2 or 15 mL were retrieved from the biobank, thawed to room temperature, and gently mixed by swirling the cryotubes. Aliquots of 0.5 mL were prepared and stored at 2°C–8°C for up to 6 h until testing.

For the diagnostic accuracy analysis, a single aliquot of each specimen was prepared for testing on a single lot of FujiLAM II. To assess inter-lot precision, four aliquots were prepared for testing with FujiLAM II using four different manufacturing lots on each of a subset of 161 specimens (644 tests) from participants with any positive TB results (AlereLAM inclusive), as further described below.

To ensure that the team carrying out the tests and reading the results could not know if a specimen was being tested multiple times or just once, a random four-digit code was assigned to each aliquot. Additionally, lot numbers of FujiLAM II assays were masked with opaque labels to ensure that the readers did not know which assay lot was being used. Urine retrieval, preparation, and labeling were conducted by one set of technicians, while a second set of technicians performed and read the tests. In this way, the technicians reading the test were fully blinded to any other test results for these specimens and to the assay lot number.

### FujiLAM II testing

After aliquoting and blinding, three technicians performed the FujiLAM II tests in batches following the numerical order of the assigned codes, typically processing six batches of four labeled aliquots per day. Prior to testing, labeled aliquots were matched with the corresponding FujiLAM II test bearing the same ID.

Testing was conducted following the manufacturer’s instructions. Briefly, urine was pipetted into the reagent tube up to the 200 µL mark using the provided pipette. The reagent tube was allowed to incubate at ambient temperature for 40 min. After incubation, one drop of the resulting solution was added to the sample well of the FujiLAM II test cartridge. Once the solution had been fully absorbed (usually within a minute), button “2” was pressed. When the “go next” indicator turned orange (approximately 15 min later), button “3” was pressed. The result was then read by inspecting the window for the appearance of a dark line within 10 min of pressing button 3.

During the 10-min reading window, two readers independently interpreted and recorded each test result according to the manufacturer’s instructions.

### Comparator tests

AlereLAM testing was performed in the original studies using fresh urine at the time of specimen collection and following the manufacturer’s instructions. Briefly, approximately 60 µL of urine was applied to the test strip and allowed to react for 25 min. The result was then read within the subsequent 10 min using the reference card provided following the manufacturer’s instructions.

For sputum smear testing, a microscopy smear with approximately 50 µL of sputum was prepared from each sputum specimen and examined for acid-fast bacilli (AFB) by fluorescence microscopy.

### Reference standard definition

All reference standard testing was performed previously, as part of the original studies (Table S2). For the main diagnostic accuracy analysis, the microbiological reference standard (MRS) included liquid and solid cultures (two sputum specimens and other specimens as clinically indicated) and molecular testing on the Xpert MTB/RIF Ultra (Xpert Ultra; Cepheid, Sunnyvale, USA) on sputum, urine, blood, and/or other specimens as clinically indicated following WHO TSS-23 guidance (10). For the inpatients in cohort 1, two sputum specimens, one blood specimen, and one urine specimen were collected for all participants, and non-sputum specimens (lymph node, ascitic fluid, gastric aspirate, and cerebrospinal fluid) were collected based on clinical indication (Table S2). For the outpatients in cohort 2, sputum specimens were collected from all participants, and non-sputum specimens were collected based on clinical indication.

Participants with any positive result (*Mycobacterium tuberculosis* complex detected) on any culture (on sputum or non-sputum specimens) and/or Xpert Ultra result of MTB detected (on sputum or non-sputum specimens) were classified as MRS positive; participants with no positive results on culture or Xpert Ultra and at least two valid negative results, including at least one negative culture result, were classified as MRS negative (Fig. S1). The diagnostic accuracy of the FujiLAM II assay was also evaluated against a sputum culture reference standard (MRS-sputum culture), including two liquid cultures; this reference standard enables comparison with the WHO targets for point-of-care tests as described in the recent target product profile (TPP) (12). Participants with any positive sputum culture were classified as MRS-sputum culture positive; participants with no positive culture and at least one negative culture result were classified as MRS-sputum culture negative. Among people with positive results on sputum culture, anyone with at least one sputum smear positive for TB was defined as having smear-positive TB; anyone with at least one negative smear and no positive smear result was classified as having smear-negative TB.

Participants with invalid or missing initial FujiLAM II results and those who had unclassifiable reference standard results were excluded from the diagnostic accuracy analysis.

### TB culture results

All specimens were processed using the N-acetyl-L-cysteine–NaOH decontamination method, and the pellet was resuspended in the same volume of phosphate-buffered saline solution. From the resuspended pellet, 0.5 mL was inoculated into a mycobacterial growth indicator tube (MGIT; BD Diagnostic Systems, Sparks, MD, USA) and incubated using the BACTEC MGIT 960 system, and approximately 0.2 mL was inoculated onto Löwenstein–Jensen media. Cultures positive for AFB were tested for *Mycobacterium tuberculosis* complex using MPT64 antigen detection (Standard Diagnostics, Republic of Korea).

### Xpert MTB/RIF Ultra results

For one sputum specimen from each participant and other non-sputum specimens collected (other than blood or urine), 0.5 mL of the resuspended pellet from culture processing was used for testing on Xpert Ultra following the manufacturer’s instructions (13).

For Xpert Ultra testing on blood specimens (cohort 1 only), a method was adapted from Banada et al. (14). Briefly, 8 mL of blood was collected into a vacutainer citrate tube and stored at 2°C–8°C for a maximum of 48 h prior to processing. The blood was processed using the NaOH–NALC method, as for culture processing, and 1 mL of the resuspended pellet was added to the Xpert Ultra sample reagent and incubated for 15 min; the specimen was then tested following the manufacturer’s instructions.

For Xpert Ultra testing on urine (cohort 1 only), about 30–40 mL of urine was first centrifuged at 3,200 × *g* for 22 min, and then the pellet was resuspended in ~1 mL of the remaining urine; Xpert sample reagent was added; and this specimen/reagent mixture was tested following the manufacturer’s instructions (15, 16).

### Statistical analysis

The sample size estimate was based on the inclusion of at least 122 participants with bacteriologically confirmed TB (positive MRS). This would enable an estimate of sensitivity of 65% (based on the WHO TPP for a non-sputum point-of-care test) (12) with a 95% CI width of ±12%.

The primary outcome was the diagnostic accuracy (sensitivity and specificity) of the FujiLAM II assay against the MRS. We calculated the point estimates of the sensitivity and specificity, together with their confidence interval at 95%, using Wilson’s method. The first FujiLAM II read was used for the diagnostic accuracy estimate. We compared the diagnostic performance of the FujiLAM II assay to the AlereLAM using Tango’s method (17). We used Cochran’s *Q* test (18) to assess precision in test positivity across the four FujiLAM II lots (lot details in Table S3). Precision testing was conducted on specimens from all participants with any microbiologically confirmed result, including any MRS-positive or Alere-LAM-positive result (Table S2). Inter-reader agreement between the first and second reads was assessed using Cohen’s kappa statistic (19). Statistical analysis was conducted using R software, version 4.5.0 (R Foundation for Statistical Computing, Vienna, Austria).

## RESULTS

### Study population

**Fig 1:**
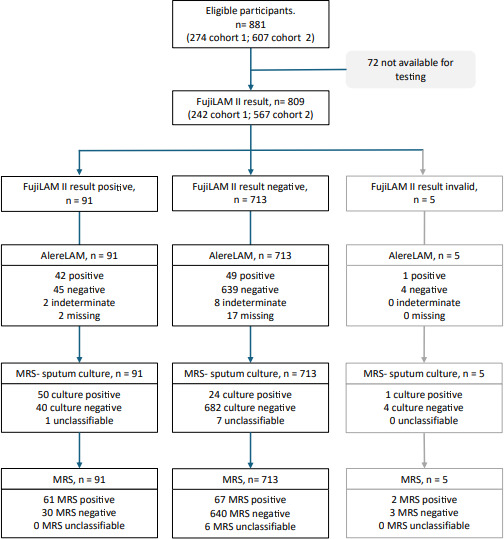
Participant flow (STARD). MRS; microbiological reference standard.

Of the 881 participants enrolled in the initial study cohorts, urine specimens from 809 participants living with HIV were retrieved and tested from across the two cohorts (242 from cohort 1 and 567 from cohort 2); urine specimens for the other 72 (8%) were not available for testing (Fig. 1). Based on the MRS definition, among 804 participants with valid FujiLAM II results, 128 (16%) were classified as having TB; 670 were classified as not having TB; and 6 were unclassifiable.

Of the 798 participants with results included in the analysis, the median age was 43 years (interquartile range 36–51); 65% were female; and 13% had a history of TB treatment (Table 1). Nearly all participants (98%) reported at least one of cough, fever, night sweats, and/or weight loss (WHO-recommended four-symptom screen), and 50% reported cough for more than 2 weeks. Overall, 78% (626/798) were on antiretroviral therapy (ART) at the time of enrollment. CD4 cell count was available for 28% of participants, of whom 91 (41%) had counts of <100 cells/µL. The prevalence of TB was 31% among inpatients (73/239) compared to 10% in outpatients (55/559). Participant characteristics by cohort are shown in Table S4.

**TABLE 1:** Baseline demographics and clinical characteristics by microbiological reference standard classification^*g*^

	All *n* = 798^*a*^		MRS positive *n* = 128		MRS negative *n* = 670	
Age, median (IQR)	43	(36–51)	41	(35–46)	43	(36–52)
Female	522	(65%)	75	(59%)	447	(67%)
History of TB^*b*^	103	(13%)	17	(13%)	86	(13%)
WHO TB symptom, any W4SS	786	(98%)	128	(100%)	658	(98%)
Cough >2 weeks	399	(50%)	60	(47%)	339	(51%)
Population						
Inpatient	239	(30%)	73	(57%)	166	(25%)
Outpatient	559	(70%)	55	(43%)	504	(75%)
HIV treatment						
On ART	626	(78%)	84	(66%)	542	(81%)
Not on ART^*c*^	172	(22%)	44	(34%)	128	(19%)
CD4 count (cells/µL)						
0–100	91	(11%)	34	(27%)	57	(9%)
101–200	59	(7%)	16	(13%)	43	(6%)
>200	71	(9%)	16	(13%)	55	(8%)
CD4 not available	577	(72%)	62	(48%)	515	(77%)
Status at 2 months						
Alive, on TB Treatment	135	(17%)	84	(66%)	51	(8%)
Alive, not on TB Treatment	438	(55%)	6	(5%)	432	(64%)
Died	27	(3%)	15	(12%)	12	(2%)
Lost to follow-up	36	(5%)	13	(10%)	23	(3%)
Status not recorded	162	(20%)	10	(8%)	152	(23%)
Xpert MTB/RIF Ultra results^*d*^						
MTB detected	115	(14%)	115	(90%)	–^*h*^	
Not detected	681	(85%)	13	(10%)	668	(100%)
TB culture results^*e*^						
Positive	84	(11%)	84	(66%)	–	
Sputum smear positive^*f*^			44	(59%)	–	
Sputum smear negative^*f*^			30	(41%)	–	
Negative	712	(89%)	42	(33%)	670	(100%)

^*a*^ Six participants had unclassifiable results on the microbiological reference standard, and five had invalid FujiLAM II results.

^*b*^ Three with unknown TB history.

^*c*^ Includes seven participants with missing ART status.

^*d*^ Includes Xpert Ultra results from any sample collected; two participants did not have an Xpert Ultra result.

^*e*^ Includes culture results from any sample collected; two participants did not have a culture result.

^*f*^ Among 74 individuals with sputum culture-positive TB.

^*g*^ ART, antiretroviral therapy; IQR, interquartile range; W4SS, WHO-recommended four-symptom screen for TB (cough, fever, night sweats, and weight loss).

^*h*^ –, not applicable.

### Diagnostic performance of FujiLAM II

Against the microbiological reference standard, in the population tested, the overall sensitivity of the FujiLAM II assay to detect TB was 48% (61/128, 95% CI 39%–56%), while specificity was 96% (640/670, 95% CI 94%–97%) (Table 2).

**TABLE 2:** Sensitivity and specificity of the FujiLAM II assay against the microbiological reference standard^*c*^

	Total	True positive	True negative	False positive	False negative	Sensitivity (%) (95% CI)		Specificity (%) (95% CI)	
All	798	61	640	30	67	48	(39–56)	96	(94–97)
Population									
Inpatients	239	38	154	12	35	52	(41–63)	93	(88–96)
Outpatients	559	23	486	18	32	42	(30–55)	96	(94–98)
Sputum microscopy (*n* = 74)^*a*^									
Smear positive	44	35	–^*d*^	–	9	80	(66–89)		
Smear negative	30	15	–	–	15	50	(33–67)		
On ART									
Yes	626	38	518	24	46	45	(35–56)	96	(94–97)
No	172	23	122	6	21	52	(38–66)	95	(90–98)
CD4 count (cells/µL) (*n* = 221)^*b*^									
<100	91	23	49	8	11	68	(51–81)	86	(75–93)
101–200	59	8	41	2	8	50	(28–72)	95	(85–99)
>200	71	5	53	2	11	31	(14–56)	96	(88–99)

^*a*^ Among sputum culture-positive individuals.

^*b*^ CD4 count results available for cohort 1 only (221 participants).

^*c*^ ART, antiretroviral therapy.

^*d*^ '–’, not applicable.

Sensitivity among inpatients was 52% (95% CI 41%–63%) as compared to 42% (95% CI 30%–55%) among outpatients. Specificity in the inpatient group was 93% (95% CI 88%–96%) as compared to 96% (95% CI 94%–98%) in the outpatient group.

Among people with sputum smear-positive TB, FujiLAM II assay sensitivity was 80% (95% CI 66%–89%), while among people with smear-negative TB, sensitivity was 50% (95% CI 33%–67%). Sensitivity was 45% (95% CI 35%–56%) among participants on ART and 52% (95% CI 38%–66%) among participants not on ART.

As compared to a sputum liquid culture reference standard, the sensitivity of the FujiLAM II was 68% (50/74, 95% CI 56%–78%), and the specificity was 94% (682/722, 95% CI 92%–96%) (Table S5).

### Comparison with AlereLAM

Among the subset of 769 people with both AlereLAM and FujiLAM II results, FujiLAM II sensitivity against the MRS was 49% (58/119, 95% CI 41%–58%) with specificity of 96% (621/650, 95% CI 94%–97%), while AlereLAM sensitivity was 35% (47/119, 95% CI 28%–45%) with specificity of 92% (601/650, 95% CI 90%–94%) (Table 3). Overall, FujiLAM II sensitivity was 13 percentage points higher (95% CI 5%–22%) than AlereLAM, and specificity was 3 percentage points higher (95% CI 1%–6%). The performance of both tests by subpopulation is reported in Table S6.

**TABLE 3:** Comparison of the sensitivity and specificity of FujiLAM II with that of AlereLAM^*c*^

	Test	Total^*a*^	TP	TN	FP	FN	Sensitivity (%) (95% CI)		Specificity (%) (95% CI)	
MRS—sputum culture	FujiLAM II	767	48	659	38	22	69	(57–78)	95	(93–96)
	AlereLAM	767	31	637	60	39	44	(33–56)	91	(89–93)
	ΔSn and ΔSp^*b*^						24	(+13 to +36)	3	(+1 to +6)
MRS	FujiLAM II	769	58	621	29	61	49	(40–58)	96	(94–97)
	AlereLAM	769	42	601	49	77	35	(27–44)	92	(90–94)
	ΔSn and ΔSp^*b*^						13	(+5 to +22)	3	(+1 to +6)

^*a*^ Twenty-nine did not have valid AlereLAM results; two did not have a sputum culture.

^*b*^ Percentage differences do not necessarily equal the differences shown here due to rounding.

^*c*^ Gray-shaded cells indicate values that are not applicable.

When a sputum liquid culture reference standard was used, including 767 people with sputum culture results, the sensitivity of the FujiLAM II increased to 69% (48/70, 95% CI 57%–78%), which was 24 percentage points (95% CI 13%–36%) higher than the sensitivity of AlereLAM (44%, 95% CI 33%–56%).

### Inter-lot precision

Among a subset of 161 participants with any TB test result positive, including any culture, Xpert Ultra, or AlereLAM-positive result, the percentage of FujiLAM II results that were positive across four manufacturing lots ranged from 37% (60/161, 95% CI 30%–45%) to 42% (67/161, 95% CI 34%–49%) (Table 4). In this subset, Cochran’s Q test showed no significant difference in positivity between lots (*Q* = 4.17, degrees of freedom = 3, *P* = 0.24), indicating that the assay performed similarly on these 161 specimens across the manufacturing lots used in this evaluation. The distribution of results by a constructed semiquantitative grade on a scale of 1–10 (Fig. S2) is shown in Fig. S3. The specifications of the lots tested are shown in Table S3.

**TABLE 4:** Positivity of the FujiLAM II assay by manufacturing lot, across 161 specimens from participants with any test result positive for TB

Lot group	FujiLAM II lot	Total	Positive	Negative	Positivity % (95% CI)	
1	Lot 11400014	161	64	97	40	(33–48)
1	Lot 11400015	161	63	98	39	(32–47)
2	Lot 11400016	161	67	94	42	(34–49)
2	Lot 11400017	161	60	101	37	(30–45)

### Inter-rater agreement

Among 772 FujiLAM II tests with two reads, the inter-rater agreement as assessed by Cohen’s kappa was 0.94 (95% CI 0.90–0.98) indicating excellent reliability between the two reads (Table S7).

## DISCUSSION

In this population of people living with HIV (both inpatients and outpatients), the FujiLAM II assay was 24 percentage points more sensitive for the detection of TB compared to AlereLAM among people with sputum culture-positive TB (69% vs 44%), and the FujiLAM II was 13 percentage points more sensitive (49% vs 35%) among people with any TB detected on a microbiological reference standard, including sputum and non-sputum specimens tested on culture and Xpert Ultra4935. The specificity of the FujiLAM II was also higher than the AlereLAM (+3%–4%) on both reference standards. An inter-lot precision analysis across four manufacturing lots showed similar positivity (from 37% to 42%) for 161 specimens from people with any microbiologically confirmed TB result, suggesting acceptable lot-to-lot precision in this new assay.

While a non-sputum point-of-care test has great potential value to improve TB detection, cost, sensitivity, and applicable patient population for use will be important considerations for widespread adoption. The AlereLAM has been recommended by the WHO since 2015 for use in people living with HIV who are seriously ill or have advanced disease (2, 20, 21). However, despite its relatively low cost (US$3.50/test) (22) and WHO endorsement, uptake across health systems has remained limited. Commonly cited barriers include budgetary constraints, regulatory delays at the national level, a narrow indication for use, lack of country-level evidence on impact, collaboration gaps between TB and HIV programs, market access and manufacturer availability, and notably, the relatively low sensitivity, even among people with low CD4 counts (22). While the FujiLAM II had better performance than the AlereLAM in this evaluation, uptake is likely to depend significantly on the price of the test, considering the WHO TPP target of ≤US$4 for a point-of-care test (or optimal price of ≤US$2) (12) and other newly available tests, such as near point-of-care tests, including the PlusLife MiniDock MTB, which is now available at less than $4/test (23, 24). Future updates to the assay, such as a urine concentration device, could help to improve the performance and potentially expand the market for this test (25).

The inter-lot precision analysis revealed similar positivity (from 37% to 42%) across four manufacturing lots (from two lot groups). The consistency in lot performance is similar to findings reported recently by Chikamatsu et al. for this FujiLAM II assay (26). For the original FujiLAM assay version, which has been discontinued, sensitivity across lots, albeit with different specimens, ranged from 33% to 74% (9). Our initial results on the FujiLAM II assay show much better agreement across lots. Nonetheless, the limited number of lot groups used in this evaluation and relatively wide confidence intervals suggest that larger studies including more lot groups will provide important evidence of the reproducibility of these new assays, especially given the past precedence of lot-to-lot variance. The WHO target product profile and the WHO TSS-23 require the use of several lots in clinical diagnostic evaluation studies but do not provide explicit methods for statistical comparison (10, 12). In contrast, Clinical and Laboratory Standards Institute guidelines recommend performing replicate testing at a 50% detection limit (C50) to compare lots (27). Given that lot-to-lot assessment is a routine process done by labs when new reagent lots are received, providing contrived samples with cultured LAM concentration that yield a known hit rate could enable laboratories to monitor lot performance effectively.

For this evaluation of the diagnostic accuracy of urine specimens on the FujiLAM II assay, we compared the performance against both a microbiological reference standard and a sputum liquid culture reference standard. The WHO technical specifications for rapid diagnostic tests to detect LAM (TSS-23) recommend evaluation against a microbiological reference standard that includes culture and molecular testing of sputum and other specimens (10). The WHO target threshold for a non-sputum point-of-care test, such as the FujiLAM II, is 65% for sensitivity and 98% for specificity of a single test when compared with sputum liquid culture (12). Since the present evaluation included only people living with HIV, rather than a general population, a more relevant comparison than the WHO TPP may be with the AlereLAM assay, the only currently available LAM test for TB and recommended only among people living with HIV, as described above.

Consistent with previous evaluations on the original FujiLAM assay version (8, 9, 28, 29) and the initial evaluation by Chikamatsu et al. (26), the FujiLAM II assay showed higher sensitivity among participants with low CD4 counts and inpatients, which are both populations at higher risk of disseminated TB where non-sputum-based diagnostics are critically needed. For example, in the evaluation of Chikamatsu, most specimens were from people living with HIV who had CD4 counts of <200 cells/mL (110/158), and the overall sensitivity reported in that evaluation was high (78.8%–79.7%). In addition, in this evaluation, the specificity of FujiLAM II was >95% overall but was lower in subgroups in which it had higher sensitivity, including inpatients and people with CD4 results of <100 cells/μL.

The FujiLAM II assay requires a 40-min incubation and several manual steps, making the overall procedure last about an hour. Nearly all tests (99.5%, Table S8) produced valid results on initial testing. When the same test was read two times independently, inter-rater agreement was very high (Cohen’s kappa of 0.94, Table S7). The assay read is performed manually, and the development of an automated, digital reading device could further improve test accuracy, as has been proposed previously (9).

This study has several limitations. Specimens were included only from people living with HIV, though the need for point-of-care TB testing includes all populations. FujiLAM II was evaluated using biobanked rather than fresh urine, while the AlereLAM was tested using fresh urine. Several reports indicate that the performance of AlereLAM and FujiLAM tests is not affected by a single freeze–thaw cycle (30). In any case, larger prospective evaluations on fresh urine from more diverse populations are needed to better characterize test performance. In this study, performance was evaluated against microbiological but not composite reference standards; future diagnostic evaluations including people with clinically confirmed TB will provide additional insight into the performance of these assays to detect TB among people with TB that is missed by other types of tests. In addition, while the results of the inter-lot precision analysis showed good agreement, this evaluation included just 161 specimens and four lots from only two lot groups, which may limit conclusions on lot-to-lot variability, as lots from similar lot groups share some intermediate materials.

Overall, these initial results suggest that the FujiLAM II assay constitutes a positive addition to the diagnostic toolbox in a frequently paucibacillary subpopulation with limited options for testing. Improvements to the assay’s sensitivity and assessing and optimizing its performance in HIV-negative persons and children, while addressing cost concerns, might further broaden its potential scope of use.

## Data Availability

All data used for the analysis are presented in the paper and its supplemental materials.

## SUPPLEMENTAL MATERIAL

Data set S1 spectrum.00620-26-s0001.csv Contains anonymized data set used for analysis in this study.

Supplemental materials spectrum.00620-26-s0002.pdf Tables S1 to S8, Figures S1 and S2, and a description of the data set shared with the manuscript have been added.

ASM does not own the copyrights to Supplemental Material that may be linked to, or accessed through, an article. The authors have granted ASM a non-exclusive, world-wide license to publish the Supplemental Material files. Please contact the corresponding author directly for reuse.
