# Supplementary material for: Diagnostic accuracy and inter-lot precision of the FujiLAM II point-of-care assay to detect tuberculosis in stored urine specimens from people living with HIV
Source: Microbiol Spectr. 2026 Jun 30;14(8):e00620-26. doi: 10.1128/spectrum.00620-26 (PMC13436352; doi:10.1128/spectrum.00620-26)
Supplement: Supplementary file 1 [file spectrum.00620-26-s0002.pdf]

Supplement to: Diagnostic accuracy and inter-lot precision of the FujiLAM II point-of-care assay to detect tuberculosis in stored urine specimens from people living with HIV

Nestor Biatu[1*], Cyrille Mbuli[1], Comfort Vuchas[1], Rita Nsamenang[1], Nadege Bonkar Chifu[1], Asonganyi Etiendem[1], Diana Kolieghu Tcheumeni[1], Angela Neh[1], Ngha Ndze Mbuh[1], Nadia Kontogianni[2], Rachel Owen[2], Ndi Norah Nyah[3], Luan Nguyen Quang Vo[4,5], Andrew Codlin[4,5], Jacob Creswell[6], Melissa Sander[1], Ana Isabel Cubas Atienzar[2]

[1]Center for Health Promotion and Research, Cameroon
[2]Liverpool School of Tropical Medicine, UK
[3]Cameroon Baptist Convention Health Services, Cameroon
[4]Friends for International TB Relief, Vietnam
[5]Karolinska Institute, Department of Global Public Health, Stockholm, Sweden
[6]Stop TB Partnership, Switzerland

Table of Contents

Table S1: Description of study cohorts 3

Table S2: List of tests performed and reference standard definitions 4

Table S3: Lot information of FujiLAM II assays used for evaluation 5

Table S4: Participant baseline characteristics and demographics, by study cohort 6

Table S5: Diagnostic accuracy of the FujiLAM II assay against sputum culture (MRS- sputum culture)....... 7

Table S6: Diagnostic accuracy of the FujiLAM II assay compared to the AlereLAM assay against the MRS . 8

Figure S1: Distribution of participants with positive MRS, AlereLAM or FujiLAM II results: a) tests included in MRS for cohort 1 participants, b) MRS results vs urine LAM results for cohort 1 participants, c) tests included in MRS for cohort 2 participants, d) MRS results vs urine LAM results for cohort 2 participants 9

Figure S2: Reference card used to read FujiLAM II results 10

Figure S3: FujiLAM test line intensity distribution by lot 10

Table S7: Agreement between first and second reads, FujiLAM II assay 11

Table S8: Test success rate, FujiLAM II, on initial and repeat test 11

Study_dataset.csv 12

Table S1: Description of study cohorts

Characteristic	Cohort 1	Cohort 2
Setting and location	Cameroon, 2 hospitals (Mbingo Baptist Hospital, Mboppi Baptist Hospital)	Cameroon, 4 health facilities (Mbingo Baptist Hospital, Mboppi Baptist Hospital, Mutengene Baptist Hospital, Baptist Health Center Nkwen)
Study population	People living with HIV ≥ 15 years old admitted for hospitalization to medical units of study hospital, regardless of symptoms	People living with HIV ≥ 15 years attending outpatient units and HIV treatment centers of study hospital with any 2 or more of WHO-recommended symptom screen (cough, fever, night sweats and/or weight loss)
Inclusion criteria	Provided informed consent<br>Willing to provide specimens for lab testing	Provided informed consent<br>Willing to provide specimens for lab testing
Exclusion criteria	On TB treatment in the last 60 days prior enrolment	On TB treatment or have received any dose of TB treatment or IPT within the 6 months prior to enrollment
Enrolment period	23-Oct-2018 to 16-Apr-2019	04-Jan-2021 to 25-Jun-2021
Protocol IRB number	IRB2018-31	IRB2020-32

Table S2: List of tests performed and reference standard definitions

Cohort 1 (inpatients)	Sputum culture reference standard (MRS-sputum culture)	Microbiological reference standard (MRS)	MRS + AlereLAM (for lot-to-Lot precision)
TB culture (liquid + solid), 2 sputum specimens (one spot and one morning)	X	X	X
TB culture (liquid + solid), non-sputum specimens[a]		X	X
Xpert Ultra, 1 sputum specimen		X	X
Xpert Ultra, 1 blood specimen		X	X
Xpert Ultra, non-sputum specimens[a]		X	X
Status at 2-month follow-up[b]			
LF-LAM (AlereLAM)			X

Cohort 2 (outpatients)	Sputum culture reference standard (MRS-sputum culture)	Microbiological reference standard (MRS)	MRS + AlereLAM (for lot-to-Lot precision)
TB culture (liquid + solid), 2 sputum specimens (one spot and one morning)	X	X	X
Xpert Ultra, 1 sputum specimen		X	X
Status at 2-month follow-up[b]			
LF-LAM (AlereLAM)			X

[a] Includes specimens collected based on clinical indication, e.g. lymph node, ascitic fluid, gastric aspirate, cerebral spinal fluid

[b]Status at 2-month follow-up was available for some participants in cohort 1 and all participants in cohort 2. AlereLAM results contributed to treatment decisions for both cohorts.

Table S3: Lot information of FujiLAM II assays used for evaluation

LOT Number	Lot Group	Catalogue Number	IFU Version (Date of Issue)	Expiry Date
Lot 11400014	Group 1	16791704	01/06/2023	Feb-26
Lot 11400015	Group 1	16791704	01/06/2023	Mar-26
Lot 11400016	Group 2	16791704	01/06/2023	Mar-26
Lot 11400017	Group 2	16791704	01/06/2023	Apr-26

Table S4: Participant baseline characteristics and demographics, by study cohort

	All[a] n = 798	Cohort 1 Inpatients n = 239	Cohort 2 Outpatients n = 559
Age, median (IQR)	43 (36, 51)	42 (35, 50)	43 (36, 52)
Female	522 (65%)	154 (64%)	368 (66%)
History of TB	103 (13%)	31 (13%)	72 (13%)
WHO TB symptom, any W4SS	786 (98%)	227 (95%)	559 (100%)
Cough > 2 weeks	399 (50%)	42 (18%)	357 (64%)
Setting			
Inpatient	239 (30%)	239 (100%)	0 (0%)
Outpatient	559 (70%)	0 (0%)	559 (100%)
Sputum microscopy[b] (n = 74)			
Smear-positive	44 (6%)	21 (9%)	23 (4%)
Smear-negative	30 (4%)	14 (6%)	16 (3%)
HIV treatment			
On ART	626 (78%)	146 (61%)	480 (86%)
Not on ART[c]	172 (22%)	93 (39%)	79 (14%)
CD4 count, cells per µL			
0-100	91 (11%)	91 (38%)	0 (0%)
101 - 200	59 (7%)	59 (25%)	0 (0%)
>200	71 (9%)	71 (30%)	0 (0%)
CD4 not available	577 (72%)	18 (8%)	559 (100%)
Status at 2 months			
Alive, on TB Treatment	135 (17%)	42 (18%)	93 (17%)
Alive, not on TB Treatment	438 (55%)	4 (2%)	434 (78%)
Died	27 (3%)	13 (5%)	14 (3%)
Lost to follow-up	36 (5%)	18 (8%)	18 (3%)
Status not recorded	162 (20%)	162 (68%)	0 (0%)
MRS			
Positive	128 (16%)	73 (31%)	55 (10%)
Negative	670 (84%)	166 (69%)	504 (90%)

[a]6 participants had unclassifiable results on the microbiological reference standard, 5 had invalid FujiLAM II results

[b]Among participants with sputum culture positive TB

[c]Includes 7 participants with missing ART status

Abbreviations: ART, antiretroviral therapy; IQR,interquartile range; W4SS (WHO-recommended four-symptom screen for TB: cough, fever, night sweats, weight loss)

Table S5: Diagnostic accuracy of the FujiLAM II assay against sputum culture (MRS- sputum culture)

	Total[a]	True Positive	True Negative	False Positive	False Negative	Sensitivity % (95% CI)	Specificity % (95% CI)
All	796	50	682	40	24	68 (56-78)	94 (92-96)
Population							
Inpatients	239	28	182	22	7	80 (63-91)	89 (84-93)
Outpatients	557	22	500	18	17	56 (40-72)	97 (94-98)
Sputum microscopy[b] (n=74)							
Smear-positive	44	35	-	-	9	80 (64-90)	-
Smear-negative	30	15	-	-	15	50 (33-67)	-
On ART							
Yes	625	33	545	29	18	65 (50-77)	95 (93-97)
No	171	17	137	11	6	74 (51-89)	93 (87-96)
CD4 Count, cells per µL							
<100	91	16	57	15	3	84 (60-96)	79 (68-88)
101-200	59	7	48	3	1	88 (47-99)	94 (83-98)
> 200	71	4	63	3	1	80 (30-99)	95 (86-99)

[a]5 results were invalid on initial testing, 8 participants did not have culture results

[b]only among participants with culture positive TB

Abbreviation: ART, antiretroviral therapy

Table S6: Diagnostic accuracy of the FujiLAM II assay compared to the AlereLAM assay against the MRS

MRS	Test	Total[a]	True Positive	True Negative	False Positive	False Negative	Sensitivity % (95% CI)	Specificity % (95% CI)
All	FujiLAM II	769	58	621	29	61	49 (41-58)	96 (94-97)
	AlereLAM	769	42	601	49	77	35 (28-45)	92 (90-94)
	ΔSn and ΔSp						13 (+5 to +22)	3 (+1 to +6)
**Population**								
Inpatient	FujiLAM II	222	36	145	12	29	55 (43-67)	92 (87-96)
	AlereLAM	222	25	139	18	40	38 (29-52)	89 (83-93)
	ΔSn and ΔSp						17 (+5 to +29)	4 (-2 to +10)
Outpatient	FujiLAM II	547	22	476	17	32	41 (30-56)	97 (95-98)
	AlereLAM	547	17	462	31	37	31 (22-46)	94 (91-96)
	ΔSn and ΔSp						9 (-3 to 22)	3 (0 to +6)
**On ART**								
Yes	FujiLAM II	603	36	503	23	41	47 (29-54)	96 (94-98)
	AlereLAM	603	25	492	34	52	32 (25-49)	94 (92-96)
	ΔSn and ΔSp						14 (+4 to +26)	2 (-1 to +5)
No	FujiLAM II	166	22	118	6	20	52 (44-71)	95 (88-96)
	AlereLAM	166	17	109	15	25	40 (25-52)	88 (83-93)
	ΔSn and ΔSp						12 (-2 to +27)	7 (+1 to +14)
**CD4 Count, cells per µL**[b]								
<100	FujiLAM II	84	22	45	8	9	71 (53-83)	85 (73-92)
	AlereLAM	84	17	46	7	14	55 (38-70)	87 (73-92)
	ΔSn and ΔSp						16 (-1 to +34)	-2 (-15 to +11)
101-200	FujiLAM II	59	8	41	2	8	50 (25-72)	95 (85-99)
	AlereLAM	59	4	38	5	12	25 (10-50)	88 (76-95)
	ΔSn and ΔSp						25 (+1 to +50)	7 (-4 to +20)
> 200	FujiLAM II	63	4	48	2	9	31 (13-58)	96 (87-99)
	AlereLAM	63	3	44	6	10	23 (8-50)	88 (76-94)
	ΔSn and ΔSp						8 (-28 to + 41)	8 (-2 to +20)

[a]29 did not have valid AlereLAM results

[b]221 from cohort 1 had valid CD4 counts, CD4 results is not avalaible for particiopants in cohort 2

Abbreviation: ART, antiretroviral therapy

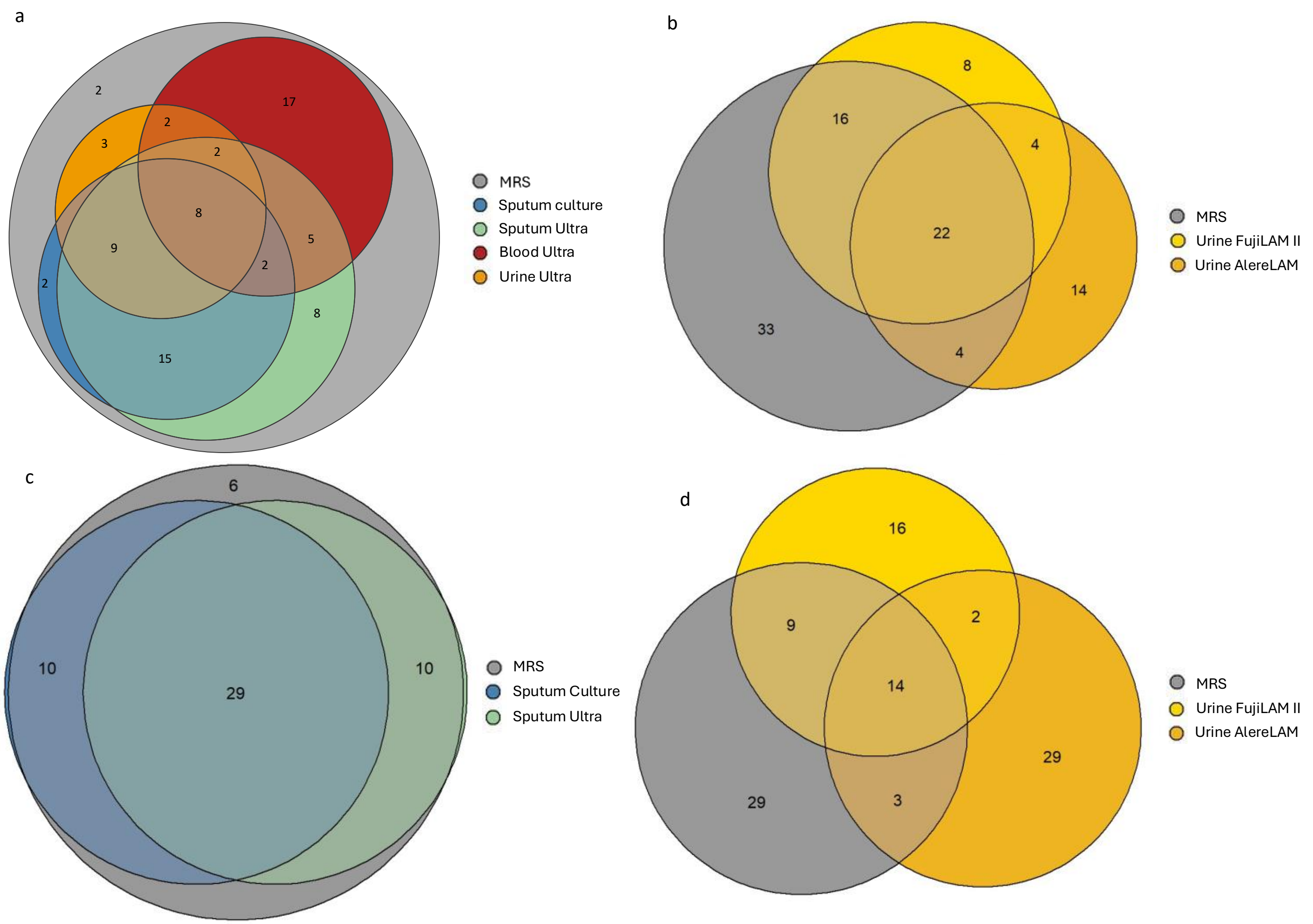

Figure S1: Distribution of participants with positive MRS, AlereLAM or FujiLAM II results:
a) tests included in MRS for cohort 1 participants, b) MRS results vs urine LAM results for cohort 1 participants, c) tests included in MRS for cohort 2 participants, d) MRS results vs urine LAM results for cohort 2 participants

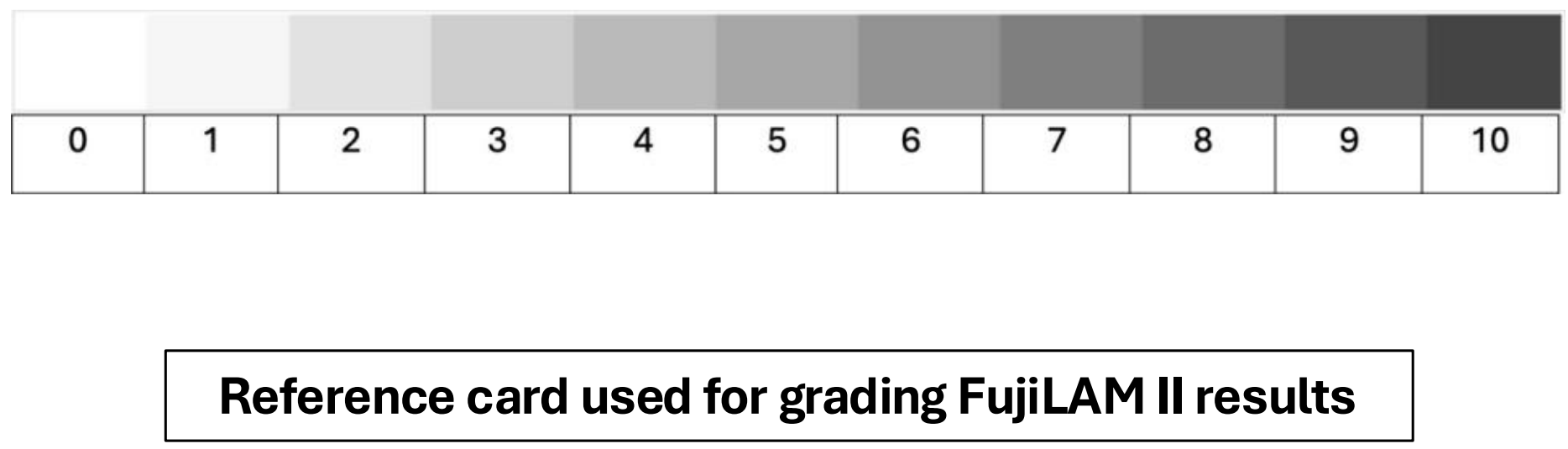

Figure S2: Reference card used to read FujiLAM II results.

Although the manufacturer did not provide a reference scale card to read results, we used one developed for this study to grade results from 0-10; as shown above negative results corresponded to a score of 0 and positive results were graded from 1 to 10, depending on the intensity of the test line.

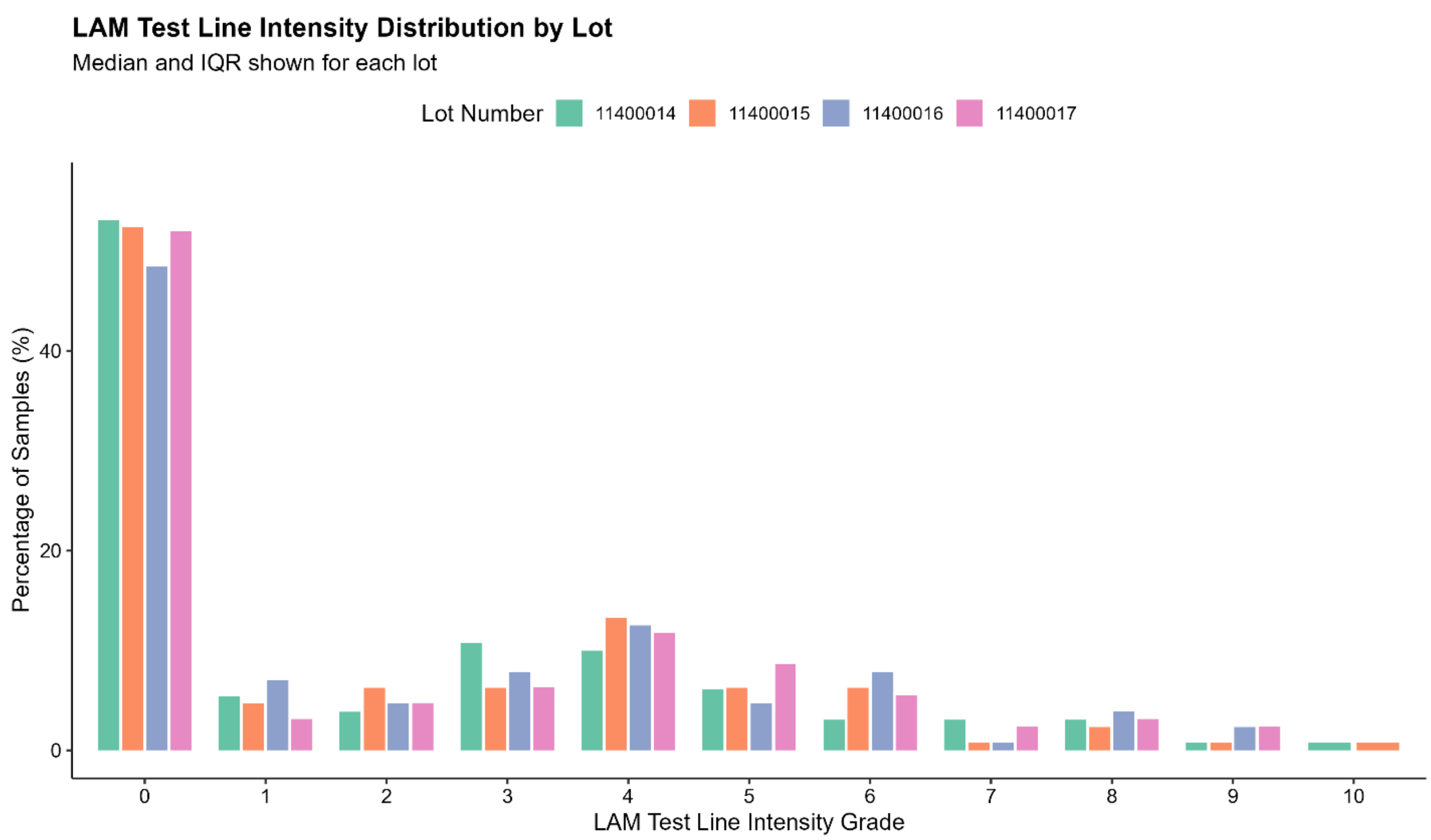

Figure S3: FujiLAM test line intensity distribution by lot

Table S7: Agreement between first and second reads, FujiLAM II assay

	Read 2 Positive	Read 2 Negative	Read 2 Invalid
Read 1 Positive	87	2	
Read 1 Negative	8	675	
Read 1 Invalid			1

K = 0.94 (95% CI: 0.90 – 0.98)

Table S8: Test success rate, FujiLAM II, on initial and repeat test

	**Overall n/N (%)**
**FujiLAM II assay**	
FujiLAM II initial invalid result	7/1369 (0.5%)
Repeat FujiLAM II result as invalid	1/7 (14.3%)

Study_dataset.csv

This file contains the anonymized dataset used for analysis in this study. Each row represents data obtained for each participants and the columns in the file include:

“study_cohort” which designates the study cohort in which the participant was enrolled

“alerelam_results” contains the AlereLAM results of participants

“fujilam2_results_LOT11400014”, “fujilam2_results_LOT11400015”, “fujilam2_results_LOT11400016”, and “fujilam2_results_LOT11400017” contains participants’ FujiLAM II results on Lots 11400014, 11400015, 11400016, 11400017 respectively.

“sputum_microscopy_smear” contains sputum microscopy smear results for participants with sputum culture positive TB

“ON_ART” contains the antiretroviral therapy status of participants at enrollment

“CD4group” contains the CD4 cell count category of participants in counts per µl

“MRS” contains the TB status of participants as described in the methods section

“OutcomeTwoMonths” contains the clinical outcome of participants at two months after enrollment.
